# Pregnancy Intentions, Maternal Behaviors, and Infant Health: Investigating Relationships With New Measures and Propensity Score Analysis

**DOI:** 10.1007/s13524-014-0359-9

**Published:** 2015-02

**Authors:** Kathryn Kost, Laura Lindberg

**Affiliations:** Guttmacher Institute, 125 Maiden Lane, 7th Floor, New York, NY 10038, USA

**Keywords:** Pregnancy intentions, Unintended childbearing, Unintended pregnancy, Maternal behaviors, Infant health

## Abstract

The premise that unintended childbearing has significant negative effects on the behavior of mothers and on the health of infants strongly influences public health policy and much of current research on reproductive behaviors. Yet, the evidence base presents mixed findings. Using data from the U.S. National Survey of Family Growth, we employ a measure of pregnancy intentions that incorporates the extent of mistiming, as well as the desire scale developed by Santelli et al. (*Studies in Family Planning, 40*, 87–100, 2009). Second, we examine variation in the characteristics of mothers within intention status groups. Third, we account for the association of mothers, background characteristics with their pregnancy intentions and with the outcomes by employing propensity score weighting. We find that weighting eliminated statistical significance of many observed associations of intention status with maternal behaviors and birth outcomes, but not all. Mistimed and unwanted births were still less likely to be recognized early in pregnancy than intended ones. Fewer unwanted births received early prenatal care or were breast-fed, and unwanted births were also more likely than intended births to be of low birth weight. Relative to births at the highest level of the desire scale, all other births were significantly less likely to be recognized early in pregnancy and to receive early prenatal care.

## Introduction

The unintended pregnancy rate in the United States has remained relatively unchanged for the past three decades, at 54 per 1,000 women ages 15–44 in both 1981 and 2008 (Henshaw 1998; Finer and Zolna 2014). In national data spanning more than two decades, more than one-third of births were reported by mothers as originating from unintended pregnancies (Mosher et al. 2012). Reports from fathers are similar (Mosher et al. 2012; Lindberg and Kost 2013). These high levels demand greater attention to the consequences of a birth resulting from unintended pregnancy.

The ability to determine whether and when to bear a child can be considered a fundamental human right and is therefore a strong basis for efforts to facilitate women’s and couples, ability to avoid unplanned pregnancies. However, research and public policy on this topic are often motivated by another equally important concern: the negative consequences associated with a birth resulting from unintended pregnancy (Institute of Medicine 2011; U.S. Department of Health and Human Services 2010). Indeed, the expansion of contraceptive insurance coverage was motivated by its potential for reducing unintended pregnancy and thereby improving public health (Institute of Medicine 2011). However, comprehensive critical reviews of research on the consequences of unintended childbearing have summarized numerous methodological challenges and concluded that the evidence base is weak and inconsistent (Gipson et al. 2008; Logan et al. 2007). In particular, U.S. studies have presented mixed evidence of the relationships between unintended pregnancy and maternal behaviors, finding weak or no effects of pregnancy intentions on key birth outcomes, such as premature delivery and low birth weight.

In this study, we examine the effect of mothers, pregnancy intentions on prenatal and postnatal health behaviors and on infant health at birth in the United States. We address two distinct issues that may underlie inconsistent or weak findings of past research: (1) the measurement and conceptualization of pregnancy intentions (the primary independent variable of interest), and (2) disentangling pregnancy intentions from demographic and socioeconomic characteristics that also affect maternal behaviors and infant health outcomes.

We explore more-nuanced measures of pregnancy intentions than used previously: an expanded version of the conventional measure that incorporates the extent of mistiming, as well as a multidimensional measure of pregnancy desire developed by Santelli et al. (2009). Additionally, we employ statistical methods as yet unused for this body of research: propensity scores. These methods offer an alternative strategy to explicitly test and adjust for observed variation in demographic and socioeconomic characteristics of mothers with differing pregnancy intentions. Our goal is to determine whether significant differences in the likelihood of performing beneficial maternal behaviors or in infant health might be attributable to underlying differences in the characteristics of women in intention status groups rather than to the direct effect of pregnancy intention itself.

## Background

### Conventional Measures of Pregnancy Intentions

The most commonly used data to measure pregnancy intentions in the United States is the National Survey of Family Growth (NSFG). Since 1973, the NSFG has enabled researchers to classify respondents, pregnancies as wanted, unwanted, or mistimed (Campbell and Mosher 2000). As part of a series of questions to document women’s pregnancy histories, respondents are asked to recall their feelings about having a baby just before they became pregnant—specifically, whether they had wanted a (or another) baby at any point in their future. If they had wanted no children or no more children, the pregnancy is classified as “unwanted.” If they wanted a baby at some point in the future, the pregnancy is classified as “wanted.”^1^ Respondents with wanted pregnancies are then asked whether the pregnancy came sooner than they would like, at about the right time, or later than they would like. Pregnancies that came sooner than preferred are classified as “mistimed.”

This retrospective “conventional” measure of pregnancy intention status was designed to obtain the most basic and essential information on a woman’s control of her reproductive life: whether she had wanted a child (or another child) just before she became pregnant and whether she became pregnant at the right time (for her). Other large-scale surveys, such as the Pregnancy Risk Assessment Monitoring System (PRAMS) and the Demographic and Health Surveys (DHS), ask the question(s) in slightly different ways, but the same categories of wanted, mistimed, or unwanted have generally been adopted.

Most often, pregnancies are characterized as either “intended” or “unintended.” Intended pregnancies are those wanted at, or sooner than, the time they occurred. Unintended pregnancies include unwanted and mistimed pregnancies. If a woman reports that she “didn’t care” whether she had a baby, the pregnancy is typically labeled as intended. Although we adopt these conventional labels, we recognize that the words “intended” and “unintended” are somewhat fraught with implied meaning of planning or intentional behavior that may or may not have occurred. Indeed, the woman is not asked whether she intended a pregnancy, but whether she wanted a baby.

Most studies of the consequences of unintended pregnancy have distinguished between two (intended or unintended) or three (intended, mistimed, or unwanted) categories of intention status. Because unintended pregnancies combine wanted pregnancies (mistimed) with those unwanted at any time, this dichotomous measure is likely too broad for investigating the impact of childbearing intentions on maternal behaviors and infant health, and may be partly responsible for the mixed findings of prior studies. The three-category measure of pregnancy intentions—with the unintended group divided into wanted but mistimed pregnancies (hereafter, “mistimed”) and unwanted pregnancies—also is likely too inclusive, combining pregnancies that were only moderately mistimed with those that were greatly mistimed. A four-category intention status measure can be constructed with responses to an additional question asked of women reporting mistimed pregnancies: how long she had wanted to wait (measured in weeks or months). Recent research has found meaningful distinctions for pregnancies that were “slightly” mistimed (by less than two years) and those that were greatly mistimed (by two or more years) (Lindberg et al. 2008; Mosher et al. 2012; Pulley et al. 2002). Thus, the four categories of this measure are (1) wanted at that time or sooner (hereafter, “intended”), (2) mistimed by less than two years, (3) mistimed by two or more years, and (4) unwanted at any time. This construct expands the conventional measure of pregnancy intentions allowing for variation in wantedness and timing.^2^

Originally designed to project fertility trends, the conventional measure of intention status from retrospective surveys has been an important population-level measure for informing policies and programs (Campbell and Mosher 2000; Finer and Kost 2011; Klerman 2000). However, concerns have been growing about its use for individual-level analyses, and numerous thoughtful critiques of this measure of childbearing intentions have been published (see Bachrach and Newcomer 1999; Klerman 2000; Luker 1999; Miller and Jones 2009; Peterson and Mosher 1999; Santelli et al. 2003). Indeed, the critiques of the conventional measure have become so numerous as to form their own body of research (see also Bachrach and Morgan 2013; Barrett and Wellings 2002; Fischer et al. 1999; Gerber et al. 2002; Higgins et al. 2012; Kaufman et al. 1997; Kavanaugh and Schwarz 2009; Kendall et al. 2005; Lifflander et al. 2007; McCormick et al. 1987; Moos et al. 1997; Petersen and Moos 1997; Poole et al. 2000; Santelli et al. 2006; Santelli et al. 2009; Stanford et al. 2000; Trussell et al. 1999; Westoff and Ryder 1977).

### Alternative Measures of Pregnancy Intentions

Conventional measures of pregnancy intention may inadequately capture gradations in attitudinal dimensions of childbearing. The measurement of wantedness using the conventional survey questions provides only whether the woman had wanted or not wanted a baby.^3^ With recognition of a need for more-refined measures, an expanded set of questions on childbearing intentions was added to more recent rounds of the NSFG (Klerman and Pulley 1999; Peterson and Mosher 1999; Mosher et al. 2012), including two questions measured on a Likert-type scale to assess *how much* women had *wanted* to avoid or have a pregnancy, and *how much* they had been *trying* to avoid or become pregnant.^4,5^ While the wanting scale is an expanded measure of the same dimension included in the conventional measure, the trying dimension is new. In essence, this question asks women to think back to before they were pregnant and assess how much effort they had put into reaching their childbearing goals. Although these two scales have been included in the NSFG since 2002, few studies have used these measures instead of, or in addition to, the conventional measure of intention (Miller and Jones 2009; Mosher et al. 2012; Santelli et al. 2009).

Beginning with the 1995 NSFG, another scaled measure was included to gauge women’s happiness when they discovered they were pregnant. Many women who experience unintended pregnancies nonetheless report high levels of happiness (Hartnet 2012; Lindberg et al. 2008; Trussell et al. 1999). This measure of happiness can be a stronger predictor of women’s behaviors during pregnancy than their reported intentions (Blake et al. 2007; Sable and Libbus 2000; Santelli et al. 2009).

From 2002 onward, the NSFG survey also asked the woman about her male partner at the time of the pregnancy and whether she had wanted to have a baby with him, building from influential research on a clinical sample by Zabin et al. (2000) finding that women expressed not wanting to get pregnant “with this partner.” Thus, how the woman felt about becoming a parent with a current partner may be yet another dimension contributing to how much she had wanted to have or avoid a pregnancy (Kroelinger and Oths 2000).

Santelli et al. (2009) sought to develop an improved multidimensional measure of unintended childbearing using the additional NSFG questions. They devised a “desire scale” by combining all the aforementioned measures: that is, the wantedness component from the conventional measure (wanted/unwanted); the three Likert-scale questions on wanting, trying, and happiness when pregnancy was discovered; and the question on whether the woman had wanted to have a baby with that partner.^6^ The desire scale parsed into seven ordinal categories had a strong relationship with pregnancy outcomes, such that women who had low levels on the desire scale were more likely to obtain an abortion than women with higher levels (Santelli et al. 2009). Timing is not part of the desire scale; but, in factor analysis, Santelli et al. (2009) identified the extent of mistiming as a unique dimension predictive of the decision to abort or continue the pregnancy.

### Testing Two Expanded Measures of Pregnancy Intentions

In this analysis, we examine whether maternal behaviors and birth outcomes differ by pregnancy intentions. We compare findings using two measures of pregnancy intentions: the conventional one expanded to four categories (intended, mistimed by less than two years, mistimed by two or more years, or unwanted), and a version of the multivariable desire scale proposed by Santelli et al. (2009).

These measures share some traits but not others. First, only the conventional measure includes the timing dimension, but it is more limited than the desire scale in its ability to capture variation in the *strength* of pregnancy wantedness. In addition, the desire scale includes information gleaned from the inclusion of several measures, including happiness about being pregnant and the woman’s attitude toward having a baby with the father.

## Previous Findings

At the population level, unintended pregnancy rates in the United States differ sharply by demographic and socioeconomic subgroup (Finer and Zolna 2014). At the individual level, pregnancy intentions are strongly related to women’s basic demographic characteristics (age, marital status, race, ethnicity, and parity) as well as socioeconomic characteristics (educational attainment, income, and poverty status) (D’Angelo et al. 2004; Hayford and Guzzo 2010; Joyce et al. 2000b; Kost and Forrest 1995; Pulley et al. 2002; Williams 1991; Williams et al. 1999). These same individual-level characteristics can also predict late recognition of pregnancy (Ayoola et al. 2009); later initiation or lower levels of prenatal care (Ayoola et al. 2010; Centers for Disease Control and Prevention 2002; Taylor et al. 2005); initiation, continuation, and exclusive use of breast-feeding after delivery (Ahluwalia et al. 2003; Centers for Disease Control and Prevention 2002; DiGirolamo et al. 2005; Jones et al. 2011; Li et al. 2005; McDowell et al. 2008; Merewood et al. 2006; Singh et al. 2007; Thulier and Mercer 2009); and poor birth outcomes, such as small for gestational age (McCowan and Horgan 2009), low birth weight (Ashdown-Lambert 2005; Blumenshine et al. 2011; Keeton and Hayward 2007), or preterm births (Afable-Munsuz and Braveman 2008; Blumenshine et al. 2011; El-Sayed et al. 2012; Keeton and Hayward 2007). Thus, the effects of pregnancy intentions on these outcomes are likely to be confounded with the effects of the mother’s background characteristics. Without an ability to randomly assign women to intention statuses—the gold standard for causal inference—researchers have used multivariate regression to control for mothers, characteristics when testing for a relationship between pregnancy intentions and maternal behaviors or infant health (Altfeld et al. 1997; Baydar 1995; Joyce et al. 2000a; Korenman et al. 2002; Kost et al. 1998a, 1998b; Marsiglio and Mott 1988; Mohllajee et al. 2007; Pulley et al. 2002; Weller et al. 1987).

### Prior Studies Relating Pregnancy Intentions to Maternal Behaviors and Infant Health

Research on the consequences of unintended childbearing dates back at least to Forssman and Thuwe’s (1966) 21-year follow-up study of 120 births in 1939–1942 to Swedish women denied abortions and matched control births born at the same hospital on the same day. The two groups differed significantly both in their background characteristics and in a range of child outcomes, including health, educational and occupational attainment, public assistance, and criminal, military, and social services records. The researchers concluded that an unwanted child is “born into a worse situation” than other children and “runs a risk of having to surmount greater social and mental handicaps than its peers” (p. 87). However, all statistical comparisons were tests of differences in outcomes for the two groups with no attempt to control for differences in background characteristics.

Similarly, Henry David and colleagues (1988) studied 220 births in 1961–1963 to women twice denied an abortion in Prague, Czechoslovakia. Births to these mothers were matched to control births whose mothers did not try to terminate the pregnancy. Further, births were matched in pairs, using age, birth order, number of siblings, and school class, and were followed into childhood. This matching strategy was an early attempt to control for the differing background characteristics underlying the intention status groups. The researchers also found that unwanted births faced significantly more disadvantages on health and school performance measures.

Following these groundbreaking studies, other researchers have investigated the impact of pregnancy intentions on maternal behaviors and infant health. In 1995, the Committee on Unintended Pregnancy, convened by the Institute of Medicine at the National Academies of Science, reviewed research to date and concluded that

The consequences of unintended pregnancy are serious, imposing appreciable burdens on children, women, men and families. A woman with an unintended pregnancy is less likely to seek early prenatal care and is more likely to expose the fetus to harmful substances (such as tobacco or alcohol). The child of an unwanted conception especially (as distinct from a mistimed one) is at greater risk of being born at low birthweight, of dying in its first year of life, of being abused, and of not receiving sufficient resources for healthy development. The mother may be at greater risk of depression and of physical abuse herself, and her relationship with her partner is at greater risk of dissolution. Both mother and father may suffer economic hardship and may fail to achieve their educational and career goals. (Brown and Eisenberg 1995:250–251).

Perhaps most importantly, the committee also concluded that, “Unintended pregnancy is not just a problem of teenagers or of unmarried women or of poor women or minorities; it affects all segments of society” (Brown and Eisenberg 1995:250). In short, this widely cited report concluded that the negative effects of unintended childbearing were not simply due to underlying maternal characteristics.

Each of the maternal behaviors and birth outcomes addressed in this article—early pregnancy recognition, early initiation of prenatal care, breast-feeding, low birth weight, and preterm delivery—has a substantial body of literature linking it with infant and child health (American Academy of Pediatrics 2012; Ayoola et al. 2009; Ayoola et al. 2010; Callaghan et al. 2006; Kramer and Kakuma 2004; MacDorman et al. 2013; McDowell et al. 2008; U.S. Department of Health and Human Services 2011). However, the relationship between pregnancy intentions and these outcomes remains less clear. In 2008, Gipson and colleagues provided an updated review of the research literature, focusing specifically on methodologically rigorous studies that attempted to control for sociodemographic background characteristics; but they found mixed evidence for the effects of pregnancy intentions on early pregnancy recognition, early prenatal care initiation, and measures of infant health at birth (Gipson et al. 2008). For example, although numerous U.S.-based studies showed an association between pregnancy intentions and delayed initiation of prenatal care, several studies also found that the relationship was diminished in multivariate analyses that included measures of the mother’s demographic and socioeconomic characteristics. However, after that review, Cheng et al. (2009) examined PRAMS data limited to births in Maryland and found that both mistimed and unwanted births were significantly less likely to initiate prenatal care in the first trimester of pregnancy, after controlling for maternal age, race/ethnicity, education, marital status, Medicaid status, and parity.

After examination of both U.S. and European studies, Gipson et al. (2008) concluded that there was consistent evidence of a relationship between pregnancy intentions and breast-feeding, but one of the five U.S.-based studies reviewed did not find a significant relationship overall (Marsiglio and Mott 1988), and one did not find significantly lower probabilities of breast-feeding among mistimed births (Kost et al. 1998b).^7^ Additionally, although many studies have found bivariate associations between pregnancy intentions and breast-feeding initiation and duration, the effects of pregnancy intentions on breast-feeding can be greatly diminished by covariates included in the analyses (Cheng et al. 2009; Joyce et al. 2000a). Similarly, a meta-analysis of the effects of pregnancy intention on low birth weight and preterm birth found consistent evidence of a bivariate relationship across studies: mistimed and unwanted births were more likely to be low birth weight and preterm than were intended births (Shah et al. 2011). Yet, among studies that included background characteristics of the mothers, only two were based on nationally representative data from the United States and examined births occurring in the 1980s and early 1990s (Kost et al. 1998b; Joyce et al. 2000a). Joyce et al. (2000a) found no association of pregnancy intentions with an infant’s risk of low birth weight; the analyses did not examine preterm births. Similarly, Kost et al. (1998b) found no significant relationship of pregnancy intention with a combined measure of preterm birth, low birth weight, or small for gestational age after maternal behaviors during pregnancy and sociodemographic characteristics were included in the model. Furthermore, a recent population-based study of births in Ireland also found no significant relationship of pregnancy intentions with low birth weight or preterm birth after adjusting for mothers, background characteristics, although births from mistimed and unwanted pregnancies were combined in one unintended category (McCrory and McNally 2013).

In the only nationally representative study to investigate the impact of pregnancy intentions on the timing of the mother’s pregnancy recognition, Kost et al. (1998a) used data from the 1988 National Maternal and Infant Health Survey and found that mothers of mistimed and unwanted births were significantly less likely to recognize that they were pregnant within the first six weeks of pregnancy than mothers of intended births, even after the researchers controlled for numerous background characteristics.

## Data and Methods

Data for this study come from pregnancy histories of women surveyed in the 2002 and 2006–2010 NSFG conducted by the National Center for Health Statistics (NCHS). The NSFG is a national probability survey of the noninstitutionalized population aged 15–44 in the United States (Groves et al. 2009; Lepkowski et al. 2006, 2013). Following recommended protocols, we pooled observations from the 2002 (*n* = 7,643) and 2006–2010 (*n* = 12,279) surveys (National Center for Health Statistics 2011). We focused on women’s behaviors during pregnancy and immediately after the birth, as well as infant health outcomes; thus, our analysis is limited to pregnancies ending in a birth. Although the conventional measure of pregnancy intention is asked for every reported pregnancy, the trying, wanting, and happiness scales are limited to pregnancies that occurred within three years of interview. Accordingly, to compare findings for the two measures of intentions, the unit of analysis is nonmultiple live births in the three years prior to the survey interview. This shorter time frame should reduce the risk of retrospective reporting bias, although we did not find evidence of bias in preliminary analyses. Women can contribute more than one birth to the analysis; 27 % of births have at least one sibling in our analytical sample. However, we included all births in the three-year window because the pregnancy histories of women interviewed in the NSFG are expected to be representative of all births in the United States near the time of the survey (Joyner et al. 2012; National Center for Health Statistics 2011). We accounted for potential autocorrelation among births with the same mother by including the mother’s unique identification code as a cluster indicator in complex survey design commands, in addition to the other design variables specified for use with the NSFG.^8^

We also limited analyses to births of mothers age 20 or older at conception. With 73 % of births to teen mothers unintended, it is difficult to parse the role of intention status from the role of age.^9^

The number of observations for analysis was 4,297, representing all singleton live births to women aged 20–44 in the periods 1999–2002 and 2004–2010.^10^

In analyses, we used the four-category conventional measure of pregnancy intentions as well as the desire scale, ranging from the lowest desire value of 0 to the highest at 6. We parsed this continuous measure into a five-category desire scale, based on quintiles of its frequency distribution.^11^ We constructed outcome measures with binary responses (no/yes) following guidelines in *Healthy People 2020* (U.S. Department of Health and Human Services 2011). We used two measures of maternal health behaviors during pregnancy: (1) mother recognized she was pregnant within the first six weeks of the pregnancy, and prenatal care was initiated in first trimester; and (2) one measure of a maternal health behavior following pregnancy: breast-feeding. We examined whether the baby was ever breast-fed for any length of time; and among those who breast-fed, whether the infant was exclusively breast-fed for at least six months (limited to births with age greater than six months at interview), and whether breast-fed for at least one year (limited to births with age greater than 12 months at interview). Finally, we examined two measures of infant health at birth: preterm delivery and low birth weight.^12^

### Analytic Strategy

We first examined whether our dependent variables—maternal health behaviors and birth outcomes—vary by demographic and socioeconomic characteristics of the mothers because these characteristics are also associated with pregnancy intentions and would be potential confounding variables. We then identified variation in the distribution of the mothers’ demographic and socioeconomic characteristics across the four intention status groups of the conventional measure and the five categories of the desire measure. These distributional differences motivated our efforts to separate the effects of pregnancy intentions on the outcomes from those attributable to the background characteristics.

Next, we employed inverse propensity (probability) weights, an adaptation of propensity score analysis. Generally, propensity score methods are used for adjusting the distribution of characteristics of two groups (a treatment and a control group) so that they are matched (“balanced”) with respect to observed characteristics that are relevant to group assignment but that also affect the outcome of interest (Austin 2011; Rosenbaum and Rubin 1983; Stuart 2010). Imbens (2000) extended the logic of propensity score methods for applications involving more than two groups by estimating group-specific propensity scores. One can apply the inverse of these probabilities to weight observations and create balanced comparison groups (McCaffrey et al. 2013). We used this method for our analysis of multiple intention status groups.

Intended births were weighted by the inverse of the propensity of having been intended; births mistimed by less than two years were weighted by the inverse of the propensity of being in that mistimed group, and so on. Inverse propensity weighting gives greater weight to observations in each intention status group that have a low probability of being in that group so that they represent a larger proportion of the births in their group in weighted analyses. The inverse weighting creates distributions of characteristics for each group that resemble the full sample, thus equating the groups.

The propensity scores used for weighting were estimated from a multinomial logistic regression, with intention status as the dependent variable (intended births were the reference category). The independent variables included in the model were all available demographic and socioeconomic measures that prior research has found to be related to intention status: age of the mother at conception (20–24, 25–29, 30–44, as well as a continuous measure of single year of age), maternal union status at conception (married, cohabiting, not in union), maternal race/ethnicity (non-Hispanic white, foreign-born Hispanic, native-born Hispanic, non-Hispanic black, non-Hispanic other races),^13^ whether the mother is foreign-born (no, yes), and whether the mother was a high school graduate at interview (no, yes). Although education measured after the birth cannot predict the pregnancy intention of the birth, we reasoned that for most mothers in our sample (age 20 or older at conception), very few who had not completed high school before age 20 would complete it after that age. We also included the respondent’s mother’s education (less than high school, high school graduate or GED, some college or more), the order of the birth (first birth, second birth, third or higher-order birth), and whether the delivery was paid for by Medicaid. We used this latter measure as a rough proxy for economic status near the time of the birth because income—and poverty status—is measured only at the time of interview and could be affected by the birth or subsequent ones.

Also included in the models were variables to account for survey implementation and other potential biases; we controlled for three periods covered by the NSFG surveys (women interviewed in 2002, 2006 to the first half of 2008, and the last half of 2008 to 2010) and the natural log of the length of recall (measured in months, from birth to mother’s date of interview).

Unlike multivariate regression used for explanatory purposes, we did not seek parsimony in the regression model used for estimating propensity scores. All measures potentially predictive of intention status were included, regardless of statistical significance. We assessed the propensity score estimation process by calculating a “standardized bias,” defined as the absolute value of the difference in means of each of the paired intention status groups divided by the standard deviation of the mean for all births (with each of the three unintended birth groups compared separately with the intended birth group). This measure is recommended in the statistical literature, and unlike standard statistical tests (e.g., *t* tests), the standardized bias is not affected by sample size (Stuart 2008, 2010). We finalized the propensity estimation model and considered the adjusted distributions of characteristics across intention status groups to be balanced after all estimates of standardized biases fell below .25 (see Tables 6 and 7 in the appendix).^14^ This process was iterative: we tested various interaction terms as well as the optimum array and form of variables. With our final model, we obtained for each observation (birth) the propensities of being intended, mistimed by less than two years, mistimed by two or more years, and unwanted. We used a separate multinomial logistic model to predict the propensity scores for categories of the desire scale (the highest desire group was the reference category).

Next, we estimated two sets of binomial logistic regression models for the relationship between pregnancy intentions and our dependent variables: maternal health behaviors and infant health at birth. We compared predicted marginal proportions obtained using the unadjusted data (using standard survey weights) with those obtained with the adjusted sample, after weighting each observation by the inverse of the propensity. For the analyses that included the propensity weights, we multiplied each observation’s inverse propensity weight by the survey weight in order to obtain unbiased effects based on the population of all births in the United States (DuGoff et al. 2013). All analyses were performed using the conventional measure of pregnancy intentions and then using the desire scale.

Observations with very low values on the propensity score obtain high weights in the inverse propensity weighted analyses. We treated observations with very high weights as outliers if the value of their weight was higher than the value at the 99th percentile of observations, and trimmed their weight to the value at the 99th percentile. Otherwise, large weights of only a few outliers can have a strong influence on the analysis (Lee et al. 2011).

All models were estimated using complex survey commands in Stata 13.0 (StataCorp 2013) and standard survey weights provided in the NSFG data. Only statistically significant differences with *p* values less than or equal to .05 are noted in the text, although not all findings are discussed.

## Results

### Dependent Variables by Demographic and Socioeconomic Characteristics

Among all births to mothers aged 20–44, most were recognized in the first six weeks of pregnancy (78 %), received prenatal care in the first trimester (90 %), and were breast-fed (71 %; Table 1). Of births that were breast-fed, 21 % were exclusively breast-fed for at least six months, and 26 % were breast-fed for at least a year (exclusively or not). Eleven percent of these singleton births were born preterm, and 7 % were low birth weight.

Compared with births to young, unmarried, and less-educated mothers, births to older, married, and more-educated mothers were more likely to have been recognized early in pregnancy, to receive early prenatal care, and to be breast-fed for any length of time, exclusively for the first six months or for at least one year. They were also less likely to have been born preterm or low birth weight.

First births were more likely to receive early prenatal care, to be breast-fed, and to be low birth weight than were second births. Third or higher-order births were less likely to be recognized early in pregnancy than second births. However, if they were breast-fed, they were more likely to be breast-fed exclusively for the first six months or to one year of age.

Finally, on every measure, births to black mothers had poorer outcomes than those to white mothers. Births to white mothers were more likely to receive early prenatal care than all other nonwhite births.

### Intention Status Groups: Distributions of Mothers, Characteristics

Table 2 shows the percentage distribution of births in the unadjusted sample before any attempt to balance the intention status groups using propensity scores. As expected, mothers of births in the four conventional intention status groups differ widely on basic demographic and life course characteristics, especially age, union status, and birth order. Statistically significant differences in the distribution of characteristics were tested with paired comparisons to the intended births (70 % of all births; Table 2). We also compared distributions for the two mistimed birth categories to investigate how they differ. Similarly, we compared greatly mistimed births and unwanted births (Table 2).

Compared with intended births, births in each of the three unintended groups have mothers who are significantly younger and less likely to be married; and who are more likely to be in a cohabiting union, to have had the delivery paid by Medicaid, and to have had a mother (the infant’s grandmother) who did not graduate from high school. Mothers of greatly mistimed births (by two or more years) or of unwanted births are also more likely to be black and are less likely to have graduated from high school than mothers of intended births. Unwanted births are also less likely to be the first birth and almost three times as likely to have been a third or higher-order birth (62 % vs. 26 % among intended births).

Not only do the three groups of unintended births differ from intended births in terms of their mother’s characteristics, but the unintended births differ from one another as well. Compared with births mistimed by less than two years, mothers of births mistimed by two or more years are more likely to be young (ages 20–24), to be black, and to have had the delivery paid by Medicaid (Table 2). They are also less likely to be married, in a union, or to have educational attainment beyond high school.

The characteristics of mothers among births mistimed by two or more years are also quite different from those of unwanted births. Unwanted births have a much higher proportion of older mothers (35 %) than do greatly mistimed births (11 %), and the mothers of unwanted births are more likely to be married or cohabiting at conception. Birth order is strongly related to being unwanted; unwanted births are much less likely to be first births and much more likely to be third or higher-order births than greatly mistimed births.

Characteristics of mothers also vary substantially across the five desire scale groups (Table 3). By design, there were identical proportions of births in each desire group (quintiles). With each successively lower desire category, group differences widened for many of the background characteristics. For example, the proportions of births whose mothers were married, white, or with the highest level of educational attainment decreased with each successively lower desire category. Similarly, the proportion of births whose mother was young (age 20–24), not in a union, black, whose delivery was paid by Medicaid, or who did not graduate from high school increased as the level of pregnancy desire decreased.

Births in the lowest desire group are more likely to be high parity and appear to be the most disadvantaged relative to births in the other groups, with higher proportions born to unmarried and nonwhite mothers. More than one-half are third or higher-order births (56 %) compared with only 32 % or fewer in all other desire groups. Delivery was paid by Medicaid for more than one-half of births in the lowest desire group (55%), less than one-half of deliveries in the next lowest desire group, and only one-fifth in the highest desire group were paid by Medicaid.

### Mothers, Characteristics After Weighting by Propensity Scores

We reexamined the distribution of the births by the mother’s demographic and socioeconomic characteristics after weighting the observations by the inverse of the propensity scores, and evaluated both the changes in and the absolute value of the standardized biases for the adjusted samples (Tables 6 and 7 in the appendix). Again, we wanted groups with similar distributions of these characteristics because these same factors are also likely to affect maternal behaviors and infant health (as shown in Table 1). We concluded that the inverse probability weights succeeded in balancing the four conventional intention-status groups (Table 6) and the five desire groups (Table 7).

### Effects of Pregnancy Intention Status Before and After Weighting by Inverse Propensity Scores

The top panel in Table 4 shows the bivariate relationship between pregnancy intentions and the seven measures of maternal behaviors and infant health with the unadjusted data (i.e., predicted proportions from logistic regressions using standard sample weights). All unintended births—whether mistimed by less than two years, mistimed by two or more years, or unwanted—are significantly less likely than intended births to have been recognized early in pregnancy and to have received early prenatal care. Births mistimed by two or more years and unwanted births are also less likely than intended births to have been breast-fed for any duration; and among those that were breast-fed, unwanted births were less likely to have been exclusively breast-fed for the first six months of life. Only unwanted births differ significantly from intended births on negative birth outcomes, with higher proportions born preterm and low birth weight.

Next, Table 4 shows the predicted proportions experiencing each outcome after inverse probability weighting. These are the proportions that we would expect to find if all intention status groups had similar distributions of demographic and socioeconomic characteristics. After weighting, many of the statistically significant differences observed in the unadjusted models are removed, particularly for mistimed births. However, greatly mistimed and unwanted births are still less likely to be recognized early in the pregnancy than intended births. Unwanted births also remain more health-disadvantaged than intended births: unwanted births are less likely to have received early prenatal care or to have been breast-fed and are more likely to have been low birth weight.

Before adjusting for propensities, the desire scale shows an almost linear relationship with the proportion of births experiencing each of the maternal behaviors, although the individual category differences are not always statistically significant (Table 5, top panel). The proportion of births recognized early in pregnancy and the proportion receiving early prenatal care both decrease as levels of desire decrease, and all these differences are statistically significant in comparison with the highest desired births. Similarly, the proportion of births breast-fed and the proportions breast-fed exclusively for the first six months of life decrease with decreasing levels of desire, although only differences in proportions at the two lowest levels of desire relative to the highest desire group are statistically significant. There were no statistically significant differences in the proportion of births born preterm between the highest level of desire and the other levels; however, births in the lowest desire category were significantly more likely to be low birth weight than births in the highest desire category (10 % vs. 5 %).

After the desire groups are balanced on their mother’s background characteristics by adjusting with the inverse propensity weights, lower levels of desire retain significant negative relationships with early pregnancy recognition and receipt of early prenatal care. However, there is no longer a statistically significant difference between the highest desire level and any of the other levels for breast-feeding behaviors and birth outcome measures.

## Discussion

Our findings support and extend research demonstrating strong demographic and socioeconomic differences in unintended childbearing. The demographic characteristics of mothers vary across intention status groups in predictable ways. Mothers of intended births are more often married and older than mothers of mistimed or unwanted births. However, births mistimed by less than two years are more similar in these life course measures to intended births than they are to births mistimed by two or more years. This conclusion is not surprising: women who identify their desired timing of fertility in such narrow terms may have engaged in a process of planning similar to that of women with intended births. In fact, they may be *more* likely to plan their births, as evinced by a stated preference for the timing. Greatly mistimed births have mothers who are younger and in less stable relationships than births in the other three groups. Unwanted births occur more often among older women and women with higher parity and are likely at a stage of life in which desired fertility is complete. Additionally, variation in the level of socioeconomic advantage across intention status groups is likely associated with the resources and ability to successfully control one’s fertility.

We found similar patterns across levels of the desire measure of intention, with the lowest desire group exhibiting demographic and socioeconomic characteristics similar to unwanted births. Our analysis indicates that the characteristics of groups defined by level of desire differ widely, with fewer married or white mothers, and more who had a delivery paid by Medicaid, as desirability of the birth decreased.

In this study and others, women’s characteristics are predictive of both beneficial maternal behaviors and the health of the infant at birth. Births to mothers who are young, unmarried, and of lower educational achievement tend to receive the lowest levels of beneficial behaviors and the highest levels of poor health at birth. These same characteristics are associated with unintended childbearing, and the research challenge is how to best separate the effects of pregnancy intentions from the effects of maternal characteristics.

Findings from prior studies using multivariate regression analyses have been mixed, suggesting that differing background characteristics of mothers may at least partially account for the relationship between pregnancy intentions and maternal behaviors and infant health outcomes. With many of these characteristics related both to pregnancy intentions and the outcomes, though, it may be difficult to know whether a regression model is specified correctly. If the model is incorrect, inferences about relationships found could be wrong or misleading, especially given the variation in characteristics across intention status groups. In fact, this variation suggests that interaction effects of each of the background characteristics with the intention status measure should be included in the model. However, few data sources include enough observations to use this strategy. We took a different approach, examining the relationships of pregnancy intentions and the outcomes after using propensity score weighting to create intention status groups more similar to one another with respect to the observed characteristics, thereby adjusting for them. A benefit of this approach is that propensity score methods are less sensitive to model specification errors than are regression models on the outcomes (Dehejia and Wahba 2002; Drake 1993; McCaffrey et al. 2013; Messer et al. 2010; Stuart 2010) which is a particularly important quality for this analysis given how widely the characteristics of intention status groups differ.

Estimated proportions from our inverse propensity weighted analyses are intended to model a counterfactual condition: what the difference between intention groups would be if they had the same propensity to land in the groups in which we find them. Thus, the estimates are intended to be representative not of what actually is observed in the population, but of what we would expect to see if mothers were not so different on their background characteristics. After we weighted the intention groups by their estimated propensity to be in that group, we found fewer statistically significant differences between intention groups and maternal behaviors and infant health. Still, evidence of an effect of pregnancy intentions remains. And findings for our two measures of pregnancy intention were similar, such that beneficial behaviors in the early stages of pregnancy were less frequent among unwanted births and those in anything but the highest desire scale group.^15^ In fact, the desirability measure corroborated the findings using the conventional measure and lends strength to the hypothesis that women’s attitudes toward the pregnancy affect their behaviors.

These findings support concerns that unwanted births and those with low levels of desirability face considerable disadvantages relative to wanted births that occurred at the time the mother desired the pregnancy. The desire scale includes a wider range of measured factors and may have a more nuanced association with the various components that affect women’s pregnancy intentions. However, the simpler conventional measure seems to yield somewhat clearer identification of the effects of pregnancy wantedness on infant health outcomes. The conventional measure detected a significant difference between intended and unwanted births in the proportion born low birth weight and the proportions breast-fed, even after adjustment for the propensities, while no significant difference was found for these measures in a comparison of the least and most desired births. Not all low-desire births are unwanted (71 %), which may partly explain why we did not observe significant effects for these outcomes. Still, we know very little about unwanted births or the contexts in which they occur. Further research should explore the characteristics and circumstances of mothers of unwanted and low-desire births to gain a better understanding of their particular constraints and hardships.

In this and many other studies, retrospective reporting of pregnancy intentions is a limitation because women may not be able or willing to recall a pregnancy from the past as having been mistimed or unwanted. However, biases could work in both directions. If women report previously unintended pregnancies as intended, differences in the outcome measures may actually be understated. On the other hand, some mothers may report an intended pregnancy as having been unintended if they face subsequent difficulties following the birth.

Our use of propensity score methods for disentangling women’s intentions from their traits has in many ways led our thinking on this topic back to where we started. Although there is value in identifying the effects of intentions among women with comparable background characteristics so that we may better understand how attitudes and desires affect behaviors, it is also important to recognize that childbearing intentions are so intimately related to the demographic stages and social conditions of women’s lives that separating the effects is difficult not only in statistical terms but also in ways that are substantively meaningful. Women who report a birth as having been mistimed or unwanted are telling researchers that they did not feel ready for a child or did not want to have another one. As scientists, we compare their behavior and infant health outcomes with those of women having intended births, but the public health goal is not to help mothers change their attitudes so that those unintended births become intended ones; the goal is to delay those pregnancies until women move into a life stage when they *do* want to have a baby—whether because they are older, are married, or have achieved the familial, educational, and occupational goals they desired. Similarly, the negative consequences for an unwanted birth can be alleviated not by convincing mothers to want the births, but by preventing the unwanted pregnancies.

These considerations of pregnancy intentions in the context of the life course are particularly relevant to health policy: if it is primarily underlying characteristics driving poorer outcomes, will programs focused on reducing unintended pregnancy help to improve maternal behaviors and infant health? We reason that the answer is yes: reported intention status as conventionally measured can capture characteristics of mothers that put their births at risk of negative consequences. In other words, women know when they are ready to become a mother and when they are not.

The consequences of a birth from an unintended pregnancy are likely to be wider than the limited measures examined in this analysis, affecting educational, career, and health trajectories as well as interpersonal relationships. With a goal of improving the health and well-being of mothers, infants, and families, public health policy should focus on efforts to provide women and men with the services and support they need to avoid unintended pregnancies and empower them to choose the time and circumstances of their childbearing.

## Figures and Tables

**Table 1:** Proportions of births with maternal behaviors and infant health at birth, by characteristics of the mother: Births to women aged 20–44 (unadjusted sample) from the NSFG 2002, 2006–2010

	Pregnancy Recognized in First 6 Weeks	Prenatal Care in First Trimester	Breast-fed (any length)	Breast-fed Exclusively for 6 Months^a^	Breast-fed for at Least 1 Year^a^	Preterm	Low Birth Weight	*n*
All Births to Mothers 20–44	0.78	0.90	0.71	0.21	0.26	0.11	0.07	4,297
Characteristics of Mother								
Age at conception								
Age 20–24	0.75*	0.85*	0.63*	0.15*	0.18*	0.13*	0.07	1,455
Age 25–29	0.77*	0.92	0.71*	0.21	0.25*	0.11	0.07	1,461
Age 30–44 (ref.)	0.82	0.93	0.78	0.25	0.32	0.09	0.06	1,381
Union status								
Married (ref.)	0.82	0.93	0.78	0.24	0.29	0.10	0.06	2,362
Cohabiting	0.72*	0.84*	0.61*	0.15*	0.19*	0.11	0.07	1,062
Not in union	0.69*	0.83*	0.56*	0.14*	0.20*	0.13	0.09*	873
Birth order								
First	0.78	0.93*	0.77*	0.20	0.23	0.11	0.08*	1,331
Second (ref.)	0.81	0.89	0.71	0.19	0.21	0.10	0.05	1,486
Third or higher	0.75*	0.88	0.67	0.25*	0.35*	0.12	0.07	1,480
Race/ethnicity								
Non-Hispanic white (ref.)	0.82	0.93	0.74	0.22	0.28	0.11	0.05	2,050
Hispanic, native-born	0.69*	0.88*	0.73	0.19	0.20	0.14	0.07	468
Hispanic, foreign-born	0.77	0.86*	0.79	0.21	0.26	0.08	0.05	635
Non-Hispanic black	0.68*	0.83*	0.51*	0.14*	0.18*	0.15*	0.13*	907
Non-Hispanic other	0.78	0.87*	0.72	0.27	0.31	0.07	0.06	237
Education								
<High school graduate (ref.)	0.74	0.83	0.54	0.19	0.24	0.12	0.09	888
High school graduate	0.79*	0.91*	0.75*	0.21*	0.26*	0.11*	0.06*	3,409
Her mother’s education								
<High school graduate (ref.)	0.73	0.85	0.66	0.18	0.22	0.11	0.06	1,315
High school graduate	0.79*	0.91*	0.66	0.25*	0.25	0.11	0.08	1,379
Some college or more	0.82*	0.93*	0.80*	0.20	0.29*	0.10	0.06	1,568

a Proportions breast-fed among births that were ever breast-fed.

* *p* < .05 (indicates a statistically significant difference in proportion between births in this group and the reference group)

**Table 2:** Percentage distribution of background characteristics and mean age of mothers of births, by intention status group: Births to women aged 20–44 (unadjusted sample) from the NSFG 2002, 2006–2010

Characteristics of Mother	Intended	Mistimed <2 Years	Mistimed 2+ Years	Unwanted	Total
All Births	0.70	0.09	0.08	0.13	1.00
Age 20–24	0.24	0.36*†	0.67*‡	0.38*	0.30
Age 25–29	0.35	0.34†	0.22*	0.27*	0.33
Age 30–44	0.41	0.30*†	0.11*‡	0.35	0.37
Married	0.73	0.62*†	0.33*‡	0.42*	0.65
Cohabiting	0.16	0.27*	0.36*‡	0.28*	0.20
Not in Union	0.11	0.12†	0.32*	0.30*	0.15
First Birth	0.36	0.30	0.39‡	0.13*	0.33
Second Birth	0.38	0.40†	0.29*	0.25*	0.35
Third Birth or Higher	0.26	0.30	0.32‡	0.62*	0.32
Non-Hispanic White	0.65	0.63†	0.45*	0.44*	0.60
Hispanic	0.18	0.22	0.22	0.25*	0.19
Native-born Hispanic	0.07	0.07	0.11	0.13*	0.08
Foreign-born Hispanic	0.11	0.16	0.11	0.12	0.11
Non-Hispanic Black	0.11	0.12†	0.25*	0.25*	0.14
Non-Hispanic Other	0.07	0.03*	0.08	0.06	0.06
Born Outside United States	0.19	0.22	0.17	0.17	0.19
Delivery Paid by Medicaid	0.29	0.39*†	0.63*	0.56*	0.36
High School Graduate	0.86	0.86†	0.74*	0.72*	0.83
Her Mother’s Education					
<High school graduate	0.24	0.33*	0.30*	0.37*	0.27
High school graduate	0.35	0.31	0.34	0.30	0.34
Some college or more	0.41	0.36	0.36	0.33*	0.39
Mean Age at Conception	28.5	26.8*†	24.0*‡	27.6*	27.9
*n*	2,784	352	455	670	4,261

*Note:* Proportions sum to 1 within intention status groups (column).

* *p* < .05 (paired *t* test vs. intended)

† *p* < .05 (paired *t* test vs. mistimed 2+ years)

‡ *p* < .05 (paired *t* test vs. unwanted)

**Table 3:** Percentage distribution of background characteristics and mean age of mothers of births, by desire scale group: Births to women aged 20–44 (unadjusted sample) from the NSFG 2002, 2006–2010

Characteristics of Mother	Highest Desire	Desire 4	Desire 3	Desire 2	Lowest Desire	All Births
Age 20–24	0.18	0.26*	0.28*	0.43*	0.42*	0.30
Age 25–29	0.35	0.38	0.31	0.31	0.26*	0.33
Age 30–44	0.46	0.36*	0.41	0.26*	0.32*	0.37
Married	0.86	0.76*	0.65*	0.48*†	0.40*	0.65
Cohabiting	0.10	0.17*	0.21*	0.29*	0.28*	0.20
Not in Union	0.04	0.07*	0.14*	0.23*†	0.31*	0.15
First Birth	0.38	0.34	0.39	0.31*†	0.21*	0.33
Second Birth	0.39	0.38	0.37	0.37†	0.23*	0.35
Third Birth or Higher	0.24	0.28	0.24	0.32*†	0.56*	0.32
Non-Hispanic White	0.70	0.63*	0.60*	0.57*†	0.49*	0.60
Hispanic	0.16	0.20	0.20	0.19	0.21*	0.19
Native-born Hispanic	0.05	0.08*	0.08*	0.08*	0.11*	0.08
Foreign-born Hispanic	0.11	0.13	0.12	0.10	0.11	0.11
Non-Hispanic Black	0.08	0.09	0.12*	0.18*†	0.24*	0.14
Non-Hispanic Other	0.05	0.08	0.08	0.06	0.05	0.06
Born Outside United States	0.19	0.20	0.22	0.16	0.15	0.19
Delivery Paid by Medicaid	0.20	0.29*	0.33*	0.48*†	0.55*	0.36
High School Graduate	0.88	0.86	0.85	0.80*	0.76*	0.83
Her Mother’s Education						
<High school graduate	0.24	0.23	0.27	0.28	0.33*	0.27
High school graduate	0.38	0.33	0.34	0.32	0.31*	0.34
Some college or more	0.37	0.44*	0.39	0.40	0.36	0.39
Mean Age at Conception	29.2	28.0*	28.3*	26.5*	27.1*	27.9
*n*	829	870	854	851	852	4,256

*Note:* Proportions sum to 1 within intention status groups (column).

* *p* < .05 (paired *t* test vs. highest desire)

† *p* < .05 (paired *t* test of desire 2 vs. lowest desire)

**Table 4:** Estimated proportions predicted by logistic regression of intention status on each outcome, unadjusted and adjusted with inverse propensity weights: Births to women aged 20–44 from the NSFG 2002, 2006–2010

Maternal Behaviors and Infant Health Outcomes	Intended	Mistimed <2 Years	Mistimed 2+ Years	Unwanted	*n*
Estimated Proportions, Unadjusted					
Recognized pregnancy in first 6 weeks	0.82	0.73*	0.66*	0.68*	4,158
Prenatal care in first trimester	0.93	0.88*	0.84*	0.83*	4,182
Breast-fed (any length)	0.74	0.77	0.64*	0.58*	4,137
Breast-fed exclusively for first 6 months	0.22	0.23	0.15	0.13*	2,531
Breast-fed for at least 1 year	0.27	0.24	0.20	0.28	2,117
Preterm	0.10	0.12	0.12	0.15*	4,184
Low birth weight	0.06	0.06	0.08	0.12*	4,184
Estimated Proportions, Adjusted With Inverse Propensity Weights					
Recognized pregnancy in first 6 weeks	0.81	0.74	0.67*	0.70*	4,158
Prenatal care in first trimester	0.92	0.89	0.89	0.81*	4,182
Breast-fed (any length)	0.71	0.73	0.65	0.62*	4,137
Breast-fed exclusively for first 6 months	0.22	0.29	0.17	0.13	2,531
Breast-fed for at least 1 year	0.26	0.31	0.22	0.29	2,117
Preterm	0.10	0.15	0.11	0.13	4,184
Low birth weight	0.06	0.06	0.09	0.11*	4,184

* *p* ≤ .05 (significant difference with intended births)

**Table 5:** Estimated proportions predicted by logistic regression of desire scale on each outcome, unadjusted and adjusted with inverse propensity weights: Births to women aged 20–44 from the NSFG 2002, 2006–2010

Maternal Behaviors and Infant Health Outcomes	Highest Desire	Desire 4	Desire 3	Desire 2	Lowest Desire	*n*
Estimated Proportions, Unadjusted						
Recognized pregnancy in first 6 weeks	0.88	0.81*	0.81*	0.70*	0.68*	4,122
Prenatal care in first trimester	0.96	0.92*	0.92*	0.87*	0.83*	4,145
Breast-fed (any length)	0.77	0.75	0.72	0.69*	0.59*	4,102
Breast-fed exclusively for first 6 months	0.26	0.21	0.20	0.19*	0.15*	2,517
Breast-fed for at least 1 year	0.26	0.27	0.24	0.25	0.28	2,104
Preterm	0.11	0.09	0.10	0.13	0.13	4,147
Low birth weight	0.05	0.04	0.07	0.07	0.10*	4,147
Estimated Proportions, Adjusted With Inverse Propensity Weights						
Recognized pregnancy in first 6 weeks	0.88	0.81*	0.79*	0.73*	0.71*	4,122
Prenatal care in first trimester	0.95	0.91*	0.91*	0.89*	0.82*	4,145
Breast-fed (any length)	0.71	0.71	0.71	0.74	0.65	4,102
Breast-fed exclusively for first 6 months	0.23	0.21	0.19	0.23	0.18	2,517
Breast-fed for at least 1 year	0.25	0.27	0.24	0.28	0.29	2,104
Preterm	0.12	0.08	0.11	0.13	0.11	4,147
Low birth weight	0.06	0.04	0.06	0.07	0.09	4,147

* *p* ≤ .05 (significant difference with highest desire births)

## Footnotes

1 The question is, “Right before you became pregnant with your (*N*th) pregnancy (which ended in [date]), did you yourself want to have another baby at any time in the future?” The four answer choices are “Yes,” “No,” “Not sure/Don’t know,” and “Didn’t care.” In the NSFG, very few respondents chose “Not sure/Don’t know” (*n* = 11 for our analysis of births; coded as missing). Those responding “Didn’t care” can be classified as intended, unwanted, or missing, depending on the focus of the analysis (*n* = 34 for our analysis; coded as intended).

2 Staff at the National Center for Health Statistics now use this four-category measure in their publications of NSFG analyses, and they advise all users of the NSFG to do so (personal communication, William D. Mosher, October 17, 2012). The Pregnancy Risk Assessment Monitoring System (PRAMS) initiated the additional question on timing in 2012.

3 There is some implied gradation of wantedness in the conventional measures, with overdue pregnancies presumed to have the highest level of desire and mistimed pregnancies presumed to reflect somewhat less desire than on-time pregnancies. However, the validity of this assumed gradation is unknown.

4 These questions were based on the psychosocial theories of Warren Miller, which posit multiple dimensions of pregnancy attitudes that affect motivations and behavior before, during, and after pregnancy (Miller 1992, 1994, 1998; Miller and Pasta 2002; Miller et al. 2004).

5 The specific wording for the wanting scale is as follows: “0 means you wanted to *avoid* a pregnancy and a 10 means you wanted to *get* pregnant. If you had to rate how much you wanted or didn’t want a pregnancy right before you got pregnant (that time), how would you rate yourself?” The wording for the trying scale is, “0 means trying hard *not* to get pregnant and a 10 means trying hard to *get* pregnant. If you had to rate how much you were trying to get pregnant or avoid pregnancy right before you got pregnant (that time), how would you rate yourself?”

6 The desire scale is calculated using the following formula: desire = (happiness/10) + (wanting/11) + (trying/11) + (want with partner/4) + on time - unwanted. Respondents who had wanted the pregnancy at the time it occurred receive 1 point (“on time” in the preceding equation) and those for whom the pregnancy was unwanted lose 1 point (“unwanted” in the equation).

7 Three of these five studies distinguished mistimed and unwanted births (Joyce et al. 2000a; Kost et al. 1998b; Taylor and Cabral 2002). The other two did not (Korenman et al. 2002; Marsiglio and Mott 1988).

8 We followed a method described by Lepkowski et al. (2013) to include multistage sample designs in complex surveys. However, estimated variances with specification of this cluster were virtually identical to those without.

9 Authors, tabulations of the pooled NSFG data.

10 A further 36 births with missing data were omitted in analyses of the conventional measure; and 41 births were missing a value for the desire scale measure (only three births were missing values for both measures).

11 Quintiles produced upper-level cutoff values of 2.69, 4.35, 5.09, 5.91, and 6.0. The substantive meaning of these values is unknown, as is that for the integer values used by Santelli et al. (2009). However, we expect that quintiles may capture underlying groupings of similar desire categories more accurately than arbitrary levels of integers.

12 Preterm is defined as a delivery occurring at or before 36 weeks of pregnancy; low birth weight is defined as ≤88 ounces or ≤2,500 grams (<5 lbs., 8 oz.).

13 Hereafter, racial groups are referred to as “white,” “black,” and “other.”

14 For issues related to assessment of balance, see Ho et al. (2007) and Stuart (2010).

15 We found a similar relationship of the conventional measure of pregnancy intentions to maternal behaviors, but not infant health, in an analysis of PRAMS data from Oklahoma (Lindberg et al. 2014).

## Appendix

**Table 6:** Standardized bias estimates for unadjusted and IPW adjusted paired samples: Intended births compared with each of the other three intention status groups

Background Characteristics of Mother	Intended vs. Mistimed <2 Years		Intended vs. Mistimed 2+ Years		Intended vs. Unwanted	
	Unadjusted	IPW Adjusted	Unadjusted	IPW Adjusted	Unadjusted	IPW Adjusted
Age 20–24	0.267	0.009	0.915	0.145	0.301	0.086
Age 25–29	0.024	0.039	0.288	0.029	0.177	0.014
Age 30–44	0.246	0.049	0.635	0.117	0.125	0.073
Married	0.234	0.031	0.813	0.172	0.620	0.131
Cohabiting	0.244	0.066	0.452	0.097	0.272	0.080
Not in Union	0.027	0.032	0.521	0.109	0.476	0.077
First Birth	0.135	0.068	0.061	0.139	0.506	0.205
Second Birth	0.045	0.078	0.189	0.052	0.262	0.054
Third Birth or Higher	0.087	0.145	0.130	0.083	0.755	0.146
Non-Hispanic White	0.042	0.116	0.389	0.141	0.415	0.148
Hispanic	0.109	0.195	0.091	0.080	0.169	0.125
Native-born Hispanic	0.008	0.101	0.119	0.069	0.184	0.128
Foreign-born Hispanic	0.141	0.151	0.008	0.038	0.047	0.042
Non-Hispanic Black	0.038	0.031	0.342	0.107	0.343	0.063
Non-Hispanic Other	0.186	0.065	0.066	0.037	0.028	0.028
Born Outside United States	0.071	0.143	0.050	0.033	0.043	0.026
Delivery Paid by Medicaid	0.199	0.027	0.686	0.188	0.547	0.207
High School Graduate^a^	0.008	0.160	0.307	0.120	0.360	0.082
Her Mother’s Education						
<High school graduate	0.197	0.141	0.134	0.156	0.283	0.141
High school graduate	0.077	0.091	0.024	0.001	0.103	0.085
Some college or more	0.114	0.047	0.105	0.148	0.171	0.052

*Notes:* We consider values greater than 0.25 to indicate substantial differences between the two groups in the distribution of the specific characteristic. Interactions included in the model to reduce bias were age by race, race by union status, parity by union status, and Medicaid by union status.

a Measured at interview, not at time of birth.

**Table 7:** Standardized bias estimates for unadjusted and IPW-adjusted paired samples: Highest-desire births compared with each of the other four desire groups

Background Characteristics of Mother	Highest Desire vs. Desire 4		Highest Desire vs. Desire 3		Highest Desire vs. Desire 2		Highest Desire vs. Lowest Desire	
	Unadjusted	IPW Adjusted	Unadjusted	IPW Adjusted	Unadjusted	IPW Adjusted	Unadjusted	IPW Adjusted
Age 20–24	0.166	0.033	0.200	0.042	0.514	0.067	0.502	0.214
Age 25–29	0.051	0.002	0.093	0.034	0.091	0.067	0.195	0.083
Age 30–44	0.220	0.032	0.108	0.008	0.429	0.001	0.311	0.132
Married	0.188	0.028	0.404	0.039	0.757	0.053	0.910	0.157
Cohabiting	0.151	0.003	0.239	0.009	0.440	0.009	0.422	0.064
Not in Union	0.071	0.037	0.244	0.059	0.464	0.076	0.674	0.126
First Birth	0.075	0.006	0.038	0.011	0.153	0.032	0.365	0.157
Second Birth	0.017	0.028	0.040	0.033	0.037	0.008	0.322	0.002
Third Birth or Higher	0.090	0.022	0.003	0.022	0.186	0.023	0.677	0.152
Non-Hispanic White	0.146	0.034	0.211	0.018	0.263	0.017	0.427	0.100
Hispanic	0.091	0.045	0.088	0.021	0.049	0.007	0.113	0.088
Native-born Hispanic	0.086	0.033	0.086	0.006	0.105	0.012	0.175	0.071
Foreign-born Hispanic	0.036	0.026	0.033	0.030	0.032	0.019	0.015	0.046
Non-Hispanic Black	0.025	0.025	0.093	0.012	0.255	0.016	0.398	0.041
Non-Hispanic Other	0.101	0.032	0.126	0.022	0.027	0.003	0.006	0.024
Born Outside United States	0.027	0.061	0.072	0.070	0.077	0.012	0.094	0.025
Delivery Paid by Medicaid	0.172	0.018	0.246	0.002	0.553	0.050	0.699	0.182
High School Graduate^a^	0.072	0.034	0.086	0.002	0.206	0.052	0.308	0.139
Her Mother’s Education								
<High school graduate	0.035	0.004	0.049	0.015	0.081	0.062	0.182	0.075
High school graduate	0.114	0.050	0.085	0.043	0.132	0.003	0.143	0.025
Some college or more	0.143	0.053	0.036	0.027	0.050	0.061	0.036	0.048

*Notes:* We consider values greater than 0.25 to indicate substantial differences between the two groups in the distribution of the specific characteristic. Interactions included in the model to reduce bias were age by race, age by parity, race by union status, parity by union status, and Medicaid by union status.

a Measured at interview, not at time of birth.
